# Robust α-synuclein pathology in select brainstem neuronal populations is a potential instigator of multiple system atrophy

**DOI:** 10.1186/s40478-021-01173-y

**Published:** 2021-05-03

**Authors:** Ethan W. Hass, Zachary A. Sorrentino, Grace M. Lloyd, Nikolaus R. McFarland, Stefan Prokop, Benoit I. Giasson

**Affiliations:** 1 Department of Neuroscience, College of Medicine, University of Florida, BMS J483/CTRND, 1275 Center Drive, Gainesville, FL 32610 USA; 2 Center for Translational Research in Neurodegenerative Disease, College of Medicine, University of Florida, Gainesville, FL 32610 USA; 3 McKnight Brain Institute, College of Medicine, University of Florida, Gainesville, FL 32610 USA; 4 Department of Neurology, College of Medicine, University of Florida, Gainesville, FL 32610 USA; 5 Department of Pathology, College of Medicine, University of Florida, Gainesville, FL 32610 USA

## Abstract

Multiple system atrophy (MSA) is an insidious middle age-onset neurodegenerative disease that clinically presents with variable degrees of parkinsonism and cerebellar ataxia. The pathological hallmark of MSA is the progressive accumulation of glial cytoplasmic inclusions (GCIs) in oligodendrocytes that are comprised of α-synuclein (αSyn) aberrantly polymerized into fibrils. Experimentally, MSA brain samples display a high level of seeding activity to induce further αSyn aggregation by a prion-like conformational mechanism. Paradoxically, αSyn is predominantly a neuronal brain protein, with only marginal levels expressed in normal or diseased oligodendrocytes, and αSyn inclusions in other neurodegenerative diseases, including Parkinson’s disease and Dementia with Lewy bodies, are primarily found in neurons. Although GCIs are the hallmark of MSA, using a series of new monoclonal antibodies targeting the carboxy-terminal region of αSyn, we demonstrate that neuronal αSyn pathology in MSA patient brains is remarkably abundant in the pontine nuclei and medullary inferior olivary nucleus. This neuronal αSyn pathology has distinct histological properties compared to GCIs, which allows it to remain concealed to many routine detection methods associated with altered biochemical properties of the carboxy-terminal domain of αSyn. We propose that these previously underappreciated sources of aberrant αSyn could serve as a pool of αSyn prion seeds that can initiate and continue to drive the pathogenesis of MSA.

**Supplementary Information:**

The online version contains supplementary material available at 10.1186/s40478-021-01173-y.

## Introduction

Multiple system atrophy (MSA) is an adult-onset progressive neurodegenerative disease that clinically presents with variable degrees of parkinsonism, cerebellar ataxia and automatic dysfunction with additional, more variable, neurological symptoms [1, 2]. MSA can be broadly subtyped into predominant cerebellar-type (MSA-C) or parkinsonian-type (MSA-P) [1, 2]. The neuropathological hallmark of MSA are glial cytoplasmic inclusions (GCIs) present in oligodendrocytes that were first identified by Gallyas silver staining [3] and later shown to be predominantly comprised of the presynaptic neuronal protein α-synuclein (αSyn) [4, 5]. Although GCIs are reportedly the most abundant type of αSyn pathology in MSA, variable and less abundant neuronal αSyn inclusions have also been described in many brain regions including the putamen, striatum, globus pallidus, substantia nigra, pons, inferior olivary nucleus, hippocampus, cerebellum and various regions of the cerebral cortex [6–16] even in prodromal cases [17].

The progressive accumulation of αSyn pathological inclusions is the hallmark of a spectrum of neurodegenerative disorders termed α-synucleinopathies, which include Parkinson’s disease and Lewy body dementia (LBD) [18, 19]. In these latter diseases, and in most other α-synucleinopathies, the vast majority of inclusions are in neurons [18, 19], which is congruent with αSyn being predominantly a neuronal brain protein [20–23]. Many in vitro, cellular and animal studies support the notion that αSyn pathology can spread throughout the CNS by a prion-like conformational templating mechanism [19, 24, 25]. However, the source of αSyn in MSA GCIs is still a highly debated conundrum, as it is unclear if αSyn is even expressed in oligodendrocytes [26]. Furthermore, there is little or no increase in αSyn expression in MSA brains compared to control, and it might even be decreased [27–29].

Using a novel series of antibodies targeting the carboxy-terminal region of αSyn, it is demonstrated that αSyn inclusion pathology is very abundant in the pontine nuclei and the inferior olivary nucleus of the medulla oblongata of MSA patients, but these neuronal αSyn inclusions have different histological properties compared to GCIs. We propose that this previously underappreciated, profuse reservoir of neuronal aggregated αSyn, could be a primary source of αSyn prion seeds which convert into a more aggressive GCI strain within oligodendrocytes that thereafter dominates prion-like transmission and progression within these glial cells.

## Materials and methods

### Generation of new αSyn monoclonal antibodies

Synthetic peptides listed in Table 1 corresponding to different amino acid stretches within the carboxyl-terminal region of αSyn were synthesized and purified by GenScript USA, Inc. (Piscataway, NJ). All peptides contained an added Cys residue at the amino-terminus that allowed for conjugation to Imject maleimide-activated mariculture keyhole limpet hemocyanin (mcKLH; Thermo Scientific, Waltham, MA). The peptides conjugated to mcKLH were used to immunize female BALB/c mice (Jackson Laboratory, Bar Harbor, ME) as previously described [30]. All procedures were performed according to the NIH Guide for the Care and Use of Experimental Animals and were approved by the University of Florida Institutional Animal Care and Use Committee. The spleens from the immunized mice were harvested and the white bloods cells were fused with mouse myeloma (Sp2/O-Ag14; ATCC, Manassas, VA) as previously described [30]. Following selection with HAT supplement (Sigma Aldrich, St. Louis, MO), all hybridoma clones were initially screened for reactivity by enzyme-linked immunosorbent assay (ELISA) using the respective peptides used for immunization.

**Table 1:** List of new antibodies described here

Antibody name	Immunization peptide	αSyn residues	Isotype
4F7	CAGPQEGILED	106–115	IgG1
2H1	CEGILEDMPVD	110–119	IgG1
5H12	CLEDMPVDPDN	113–122	IgG1
4F11	CDMPVDPDNEAY	115–125	IgG1
3A12	CPVDPDNEAYEMPS	117–129	IgG1

Shown are the synthetic peptides used for mouse immunization and their corresponding amino acid  residue localization in human αSyn. The isotype of each antibody is included

Antibody isotypes were determined using a mouse monoclonal isotyping kit (Millipore Sigma, Burlington, MA).

### Enzyme-linked immunosorbent assay (ELISA)

96-well ELISA plates (Corning Life Sciences, Corning, NY) were coated with 100 ng peptide in 100 µL PBS per well using the peptide used for immunization (see Table 1). Wells were washed with PBS and blocked with PBS/5% fetal bovine serum (FBS). Primary antibodies were added to blocking solution and incubated at room temperature. After PBS washes, plates were incubated with horseradish peroxidase-conjugated anti-mouse antibody (Jackson Immuno Research Labs, West Grove, PA) in 5% FBS/PBS for an hour. Plates were washed with PBS and 3,3′,5,5′-tetramethylbenzidine (TMB substrate, Thermo Fisher Scientific, Waltham, MA) was added to each well. The reactions were stopped by adding 0.5 M HCl and the optical density was measured at 450 nm with a plate reader.

### Immunohistochemistry of human brain tissue

Formalin-fixed brain samples of patients with LBD, MSA, and controls were provided by the University of Florida Neuromedicine Human Brain and Tissue Bank (UF HBTB) following institutional regulations. Postmortem diagnoses of LBD, MSA, AD neuropathological change, and other changes were made according to current guidelines and criteria proposed by the National Institute of Aging-Alzheimer’s Association [31], the Dementia with Lewy Bodies Consortium [32], and the Neuropathology Working Group on MSA [2]. See Table 2 for details on human cases used for this study.

**Table 2:** Summary of cases used in this study

Case	Clinical diagnosis	Primary pathological diagnosis	Secondary pathological diagnosis	Thal	Braak	CERAD	Gender	Age
Control 1	Normal	AD low		1	II	none	M	88
Control 2	Normal	PART		0	II	none	F	72
Control 3	Normal	No significant pathological findings		0	0	none	F	55
Control 4	Normal	Remote micro-infarcts and calcifications		0	0	none	M	59
MSA-1	MSA-C	MSA		0	0	none	F	67
MSA-2	MSA-P	MSA	AD intermediate	1	III	moderate	M	71
MSA-3	MSA-P	MSA	AD low	1	I	none	F	60
MSA-4	MSA-P	MSA	PART	0	II	none	M	77
MSA-5	MSA-C	MSA	AD low; CAA	3	I	sparse	M	71
MSA-6	MSA-P	MSA	AD low; CAA	3	I	sparse	F	68
MSA-7	MSA-C	MSA	AD intermediate	3	III	sparse	M	59
MSA-8	MSA-P/C	MSA	PART; CAA	0	I	none	F	66
MSA-9	MSA-P	MSA		0	0	none	F	66
MSA-10	MSA-P/C	MSA		0	0	0	M	53
LBD-1	DLB	LBD diffuse neocortical	AD intermediate; CAA	5	IV	moderate	M	67
LBD-2	DLB	LBD diffuse neocortical	AD high; CAA; LATE (stage 1)	5	V	frequent	F	68
LBD-3	DLB/AD	LBD diffuse neocortical	AD intermediate; CAA; LATE (stage 2)	3	V	frequent	M	83
LBD-4	DLB	LBD diffuse neocortical	AD intermediate; CAA; ARTAG	4	III	sparse	F	67

Listed are the clinical and pathological diagnoses, the sex, age at death and Thal, Braak and CERAD ratings

*AD* Alzheimer’s disease, *ARTAG* aging related tau astrogliopathy, *CAA* cerebral amyloid angiopathy, *DLB* dementia with Lewy body, *LATE* limbic-predominant age related TDP-43 encephalopathy, *LBD* Lewy body disease, *MSA-C* multiple system atrophy with predominant cerebellar ataxia, *MSA-P* multiple system atrophy with predominant parkinsonism, *PART* primary age-related tauopathy

For immunohistochemistry (IHC), paraffin-embedded tissue on slides were rehydrated in xylene and a series of ethanol solutions (100%, 90%, and 70%). For antigen retrieval, slides were treated with 70% formic acid for 20 min at room temperature and after extensive washing placed in a steam bath for 60 min in a solution of modified citrate buffer (Target Retrieval Solution Citrate pH 6; Agilent, Santa Clara, CA). Endogenous peroxidases were quenched by submerging slides in PBS solutions with 1.5% hydrogen peroxide and 0.005% Triton-X-100. After washing, slides were blocked in 2% FBS/0.1 M Tris, pH 7.6 and were incubated in primary antibody overnight at 4 °C. After washes with 0.1 M Tris, pH 7.6, a mixture of biotinylated secondary antibody (Vector Laboratories; Burlingame, CA) and ImmPRESS polymer secondary antibody (Vector Laboratories; Burlingame, CA) were similarly diluted in block solution and applied to sections for 1 h at room temperature. An avidin–biotin complex (ABC) system (Vectastain ABC Elite kit; Vector Laboratories, Burlingame, CA) was used to enhance detection of the immunocomplexes, which were visualized using the chromogen 3,3′-diaminobenzidine (DAB kit; KPL, Gaithersburg, MD). Tissue sections were counterstained with hematoxylin (Sigma Aldrich, St. Louis, MO). Slides were dehydrated in ethanol solutions (70%, 90%, and 100%) and xylene before they were covered with Cytoseal (Thermo Fisher Scientific, Waltham, MA). Slides were digitally scanned using an Aperio Slide Scanner AT2 instrument (40X magnification; Aperio Technologies Inc., Vista, CA).

Other mouse monoclonal antibodies used for IHC included anti-ubiquitin antibody Ubi-1 (Thermo Fisher Scientific, Waltham, MA), anti-p62/sequestrosome-1 (p62) antibody 5G3 [33], and C-terminal αSyn 94-3A10 specific for residues 130–140 [34].

For double labelling, the procedures were similar, but the steam bath in modified citrate buffer retrieval was performed prior to formic acid treatment. Rabbit anti-neurofilament light chain (NFL) (C28E10; Cell Signaling Technology) or rabbit anti-microtubule-associated protein 2 (MAP2) (4542; Cell Signaling Technology) antibodies were applied overnight with anti-αSyn 5H12 antibody. After the DAB reaction, the tissue was rinsed and ImmPRESS anti-rabbit conjugated to alkaline phosphatase (Vector Laboratories) was applied for 1 h. After washes, the tissues sections were incubated in 0.1 M Tris, pH 8.45 and labeling was visualized with Vector Red substrate (Vector Laboratories). Tissue sections were dehydrated and mounted as described above but without hematoxylin counterstain.

### Gallyas silver staining

For silver staining, paraffin-embedded tissue on slides were rehydrated in xylene and series of ethanol solutions (100%, 90%, and 70%) followed by a washes in dH_2_O. Sections were incubated in 5% periodic acid for 5 min, washed twice in dH_2_O for 5 min and placed in alkaline silver iodide (4% sodium hydroxide, 10% potassium iodide and 0.035% silver nitrate in 100 mL dH_2_O) for 1 min. Following a 10 min wash step in 0.5% acetic acid, the sections were placed in developer solution for 5–10 min until desired staining was achieved (Stock solution I: 5% sodium carbonate in water; Stock solution II: 0.2% ammonium nitrate, 0.2% silver nitrate, 1% tungstosilicic acid in water; Stock solution III: 0.2% ammonium nitrate, 0.2% silver nitrate, 1% tungstosilicic acid, 0.73% formaldehyde in water; 3 volumes of stock solution II added to 10 volumes of stock solution I followed by 7 volumes of stock solution III). Subsequently, sections were washed in 0.5% acetic acid for 3 min, rinsed in dH_2_O for 5 min and incubated in 0.1% gold chloride for 5 min. After a 5-min rinse in dH_2_O, sections were incubated in 1% sodium thiosulphate for 5 min and rinsed in tap water. Sections were counterstained with nuclear fast red (Thermo Fisher Scientific, Waltham, MA) according to manufacturer’s instructions. Slides were dehydrated in ethanol solutions (70%, 90%, and 100%) and xylene before they were covered with Cytoseal (Thermo Fisher Scientific, Waltham, MA). Slides were digitally scanned using an Aperio Slide Scanner AT2 instrument (40X magnification; Aperio Technologies Inc., Vista, CA).

### Sequential biochemical fractionation of human nervous tissue

White matter from the cerebellum and pons of MSA (MSA-1, -8, and -9) and control (control-1, -2, and -3) patients were used. Tissues were homogenized with 3 mL per gram of tissue with high salt (HS) buffer (50 mM Tris, pH7.5, 750 mM NaCl, 20 mM NaF, 5 mM EDTA) with a cocktail of protease inhibitors (1 mM phenylmethylsulfonyl fluoride and 1 mg/mL each of pepstatin, leupeptin, N-tosyl-L-phenylalanyl chloromethyl ketone, N-tosyl-lysine chloromethyl ketone and soybean trypsin inhibitor). The tissue homogenates then underwent sedimentation at 100,000 × g for 30 min and the supernatants were removed and kept as the HS fraction. Pellets were re-extracted in 3 mL per gram of tissue with HS buffer with 1% Triton X-100 (HS/T buffer) and centrifuged at 100,000 × g for 30 min. The supernatants were removed and kept as the HS/T fraction. The pellets were then homogenized in 3 mL per gram of tissue with HS buffer/1% Trition X-100 with 1 M sucrose and centrifuged at 100,000 × g for 30 min to float the myelin, which was discarded. Pellets were homogenized in 2 mL per gram of tissue with radioimmunoprecipitation assay (RIPA) buffer (50 mM Tris, pH 8.0, 150 mM NaCl, 5 mM EDTA, 1% NP-40, 0.5% sodium deoxycholate, 0.1% SDS) plus protease inhibitors and centrifuged at 100,000 × g for 30 min. Supernatants were removed and kept as the RIPA fraction. Pellets were then homogenized in 1 mL per gram of tissue with 2% SDS/4 M urea by probe sonication, and centrifuged at 100,000 × g for 30 min and supernatant was kept as the SDS/U fractions. Protein concentrations of all fractions were determined by BCA assay using bovine serum albumin (BSA; Pierce, Rockford, IL) as a standard. SDS sample buffer was added to the fractions which were incubated for 10 min at 100 °C (HS and HS/T fractions) or at room temperature (SDS/U fraction only). Equal amounts of protein (10 μg for each fraction) were resolved by SDS-PAGE and analyzed by immunoblot.

### Recombinant synuclein proteins

Human and mouse αSyn, human β-synuclein (βSyn), human γ-synuclein (γ Syn) and 1–129, 1–125, 1–122, 1–119 and 1–115 carboxy-truncated human αSyn were expressed using the bacterial expression plasmid pRK172 in BL21 (DE3) *E. coli* (New England Biolabs Inc). The proteins were purified using size exclusion chromatography followed by Mono Q anion exchange chromatography as previously described [35].

### Immunoblotting analyses

Protein samples were resolved by SDS-PAGE on 15% SDS–polyacrylamide gels. The proteins were then electrophoretically transferred onto 0.2 μm pore size nitrocellulose membranes (Bio-Rad, Hercules, CA) in carbonate transfer buffer (10 mM NaHCO_3_, 3 mM Na_2_CO_3_, pH 9.9) [36] with 20% methanol with a constant current of 255 mA for 75 min. Membranes were washed with Tris-buffered saline (TBS), blocked with 5% milk/TBS and incubated overnight at 4 °C with primary antibodies. Following washing, blots were incubated with horseradish peroxidase conjugated goat anti-mouse antibody (Jackson Immuno Research Labs, West Grove, PA) diluted in 5% milk/TBS for 1 h. Following washing, the labeled protein bands were visualized by chemiluminescence using Western Lightning Plus ECL reagents (PerkinElmer Life Sciences, Waltham, MA) and with a GeneGnome XRQ imager (Syngene, Frederick, MD).

## Results

**Fig. 1:**
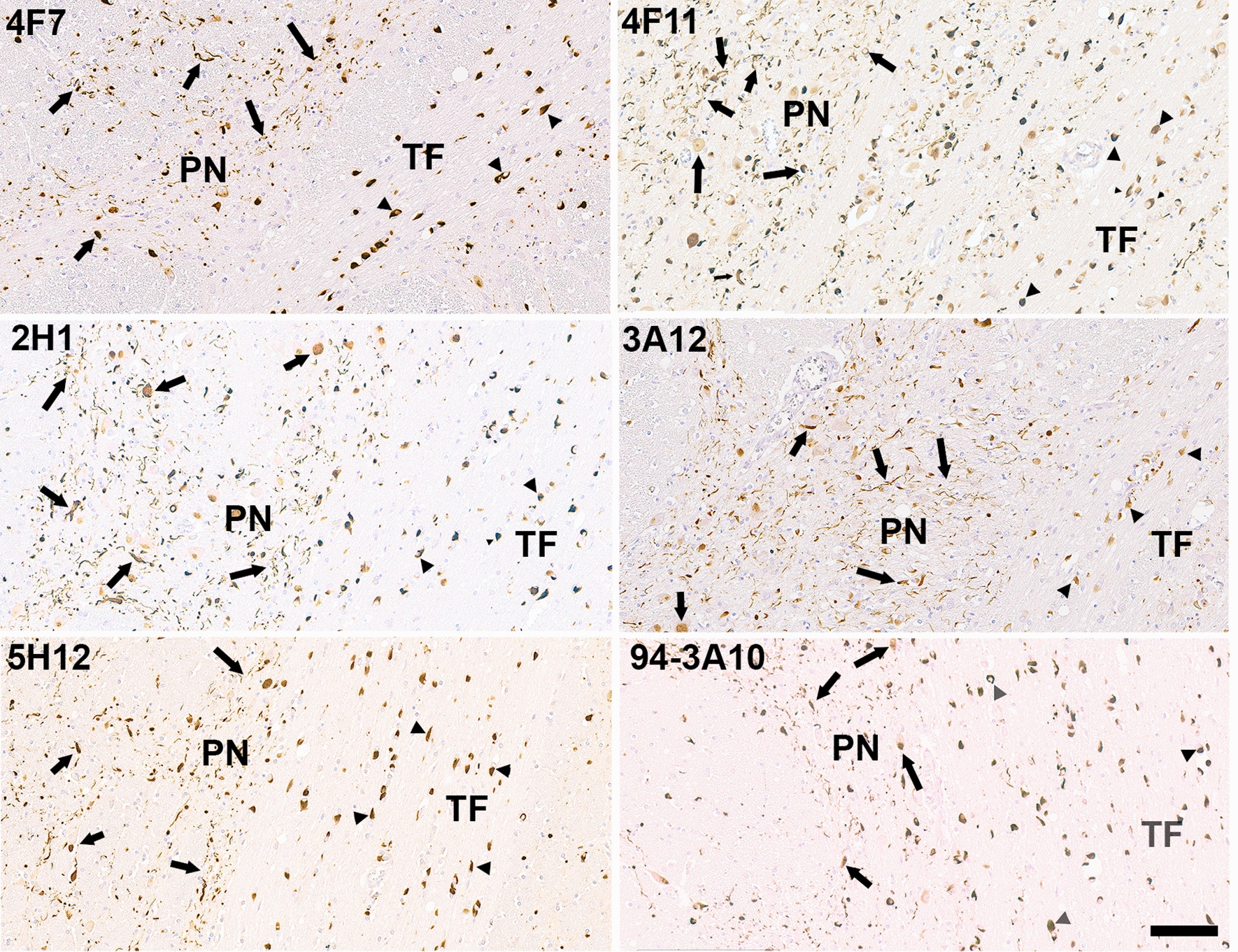
IHC staining of pontine tissue sections from an MSA patient with the various αSyn antibodies demonstrating the abundance of αSyn neuronal and neuritic pathology (arrows) in the pontine nuclei (PN) compared to GCIs (arrowheads) in the transverse fibers (TF). Tissue sections were stained with the αSyn antibodies indicated in the top left corner. All sections were counterstained with hematoxylin. Scale bar = 100 μm

**Fig. 2:**
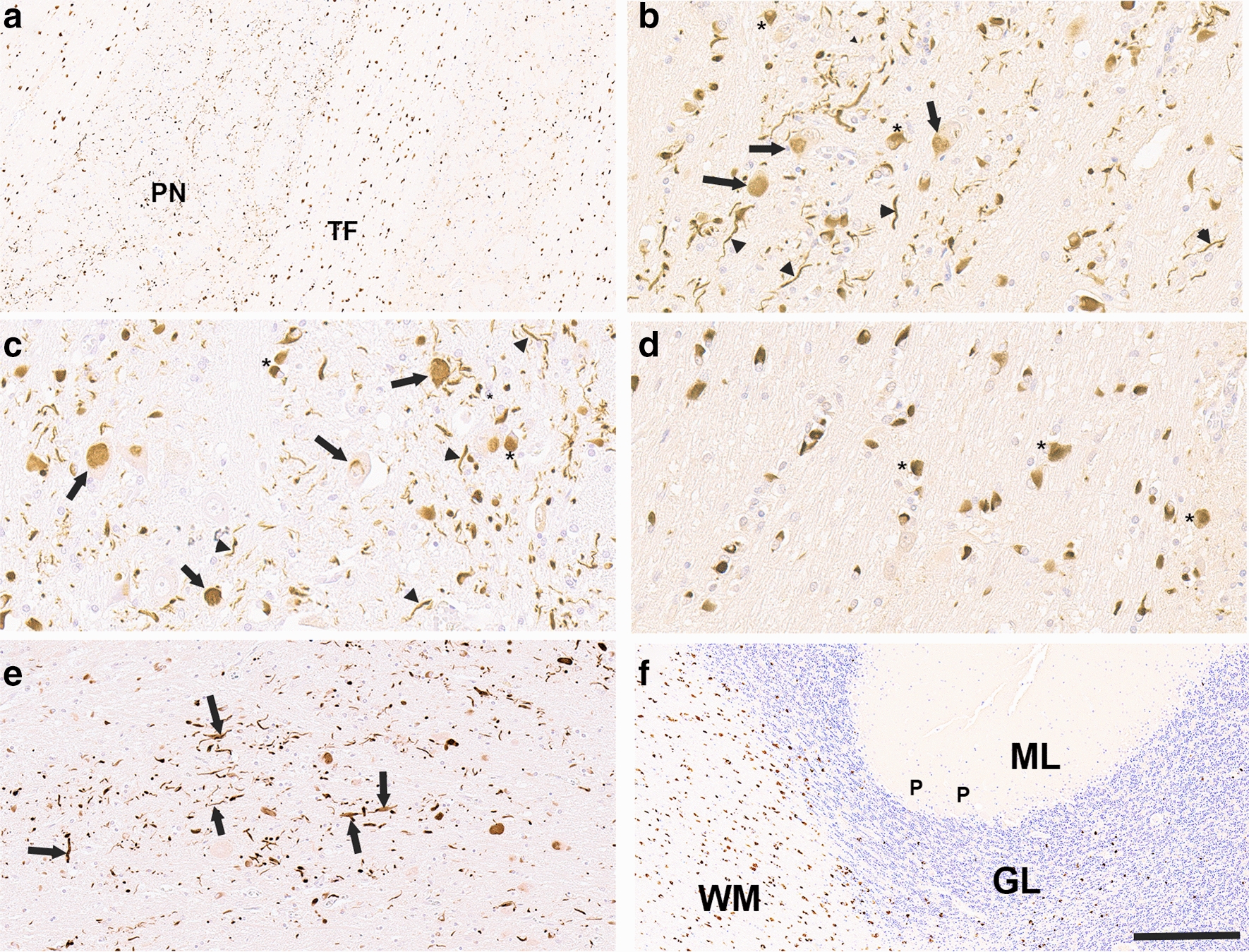
Abundant neuronal αSyn inclusion pathology in the pontine nuclei of MSA patients. **a** Low magnification image showing abundant αSyn inclusion pathology in pontine nuclei (PN) and transverse fibers (TF) within the pons with 5H12 antibody. **b**, **c** Higher magnification showing that αSyn pathology with antibodies 5H12 (**b**) and 2H1 (**c**) in the pontine nuclei is predominantly within neuronal cell bodies (arrows) and processes (arrowheads) with a few GCIs (asterisks). **d** In the transverse fibers of the pons, αSyn is predominantly in GCIs (asterisks) as stained with 5H12 antibody. (**e**) Abundant αSyn neuroaxonal pathology (arrows) was also observed in the middle cerebellar peduncle stained with 5H12 antibody. (**f**) In the cerebellum, αSyn inclusions are all in the form of GCIs predominantly in the white matter (WM), but occasionally within the granular layer (GL) stained with 5H12 antibody. Neurons of the granular layer and Purkinje neurons (P) at the boundary with the  molecular layer (ML) do not have αSyn inclusion. In **a**, **b**, **d**–**f** sections were stained with 5H12 antibody. Antibody 2H1 was used in **c**. Sections were counterstained with hematoxylin. Scale bar = 300 μm in **a**, **f**; 120 μm in **e**; 60 μm in **b**–**d**

αSyn carboxy-terminal truncation, which occurs due to a spectrum of various biological cleavage events, is the predominant post translational modification associated with the formation of αSyn pathological inclusions [37]. This type of modification can dramatically promote the formation of αSyn pathological inclusions [37]. Aiming at better understanding the relationship between these different modifications of αSyn, and pathological inclusions, we generated a novel series of monoclonal antibodies with epitopes targeting various regions within the carboxy-terminal domain of αSyn. The antibodies used in studies here are summarized in Table 1 and Additional file 1: Fig. S1. Starting with a larger series of antibodies, a subset was identified that revealed extensive abundant, and consistent neuronal pathology in the pontine nuclei across the MSA patient brains used here, as shown in representative images (Fig. 1; Table 2). These antibodies also labeled nigral Lewy bodies, cortical Lewy bodies and Lewy neurites in LBD patients (Additional file 1: Fig. S2) and GCIs in MSA patients (Additional file 1: Fig. S3), but the abundance of inclusions in neuronal cell bodies and surrounding processes in the pontine nuclei revealed by these antibodies was remarkable (Figs. 1, 2). No pathological inclusions were observed in control patients (Additional file 1: Fig. S4; data not shown). The epitopes for these antibodies were further mapped and characterized with recombinant αSyn carboxy-truncated proteins and the related proteins βSyn and γSyn (Additional file 1: Fig. S5) with findings summarized in Additional file 1: Fig. S1. Overall, the epitopes for these antibodies spanned different regions on the carboxy-terminal region of αSyn. These antibodies were used to stain various other brain regions of MSA patients including the midbrain, putamen, caudate, globus pallidus, and cerebellum that typically have abundant GCIs and less abundant neuronal αSyn inclusions [6, 7, 9]. Consistent with these previous studies, the predominant forms of αSyn inclusions observed in these regions were GCIs with some sparse neuronal inclusions observed in the midbrain and basal ganglia (Additional file 1: Fig. S3; data not shown).

**Fig. 3:**
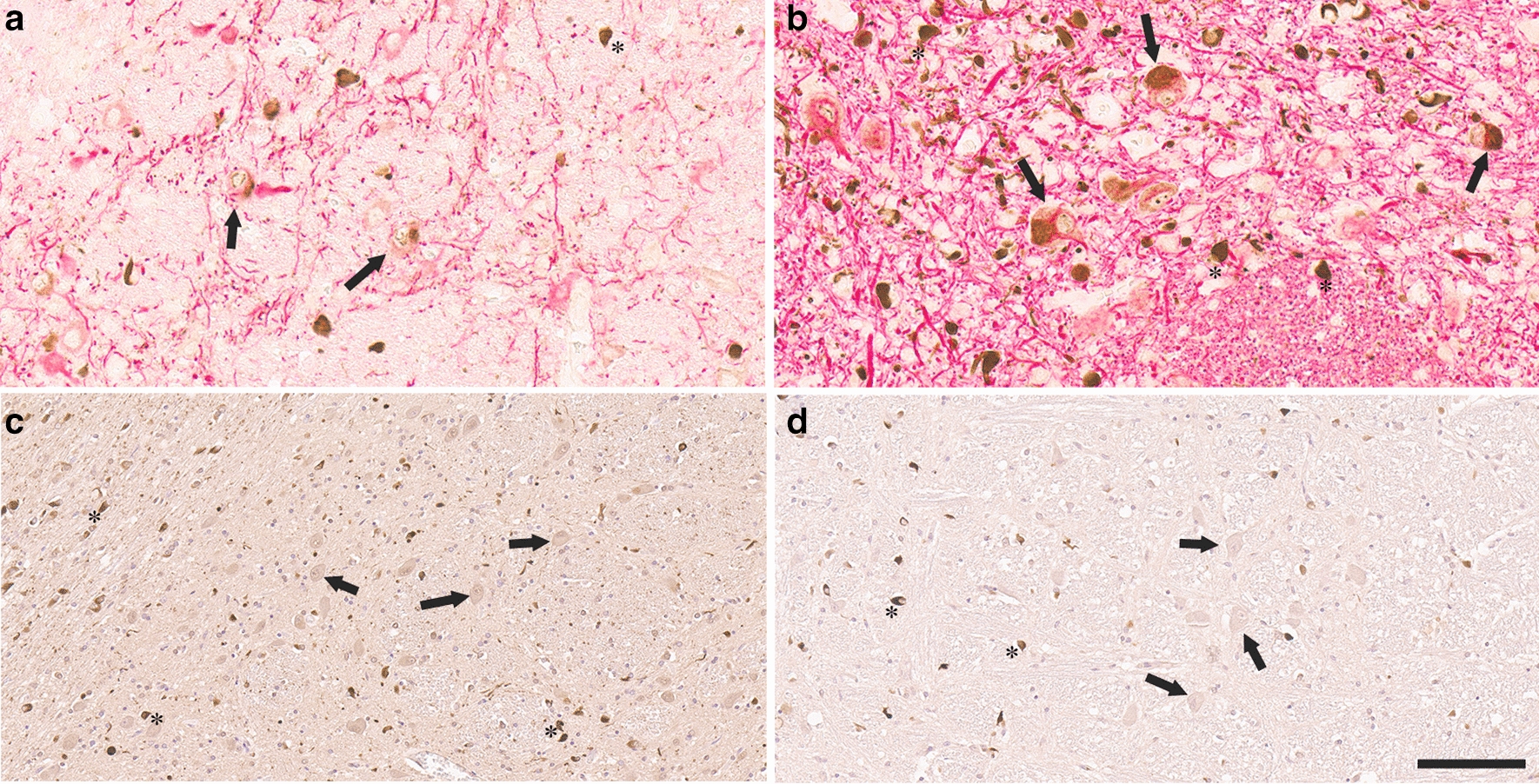
Characterization of neuronal inclusions in the pontine nuclei of MSA patients. **a** Double staining of the pons of an MSA patient with anti-MAP2 (red) and anti-αSyn 5H12 (brown) antibodies. **b** Double staining of the pons of an MSA patient with anti-NFL (red) and anti-αSyn 5H12 (brown) antibodies. **c** IHC with anti-ubiquitin antibody Ubi-1. **d** IHC with anti-p62 antibody 5G3. In **c** and **d**, the tissue sections were also counterstained with hematoxylin. Arrows depict neuronal cell bodies within the pontine nuclei and asterisks highlight GCIs. Scale bar = 60 μm in **a**, **b** and 120 μm in **c**, **d**

**Fig. 4:**
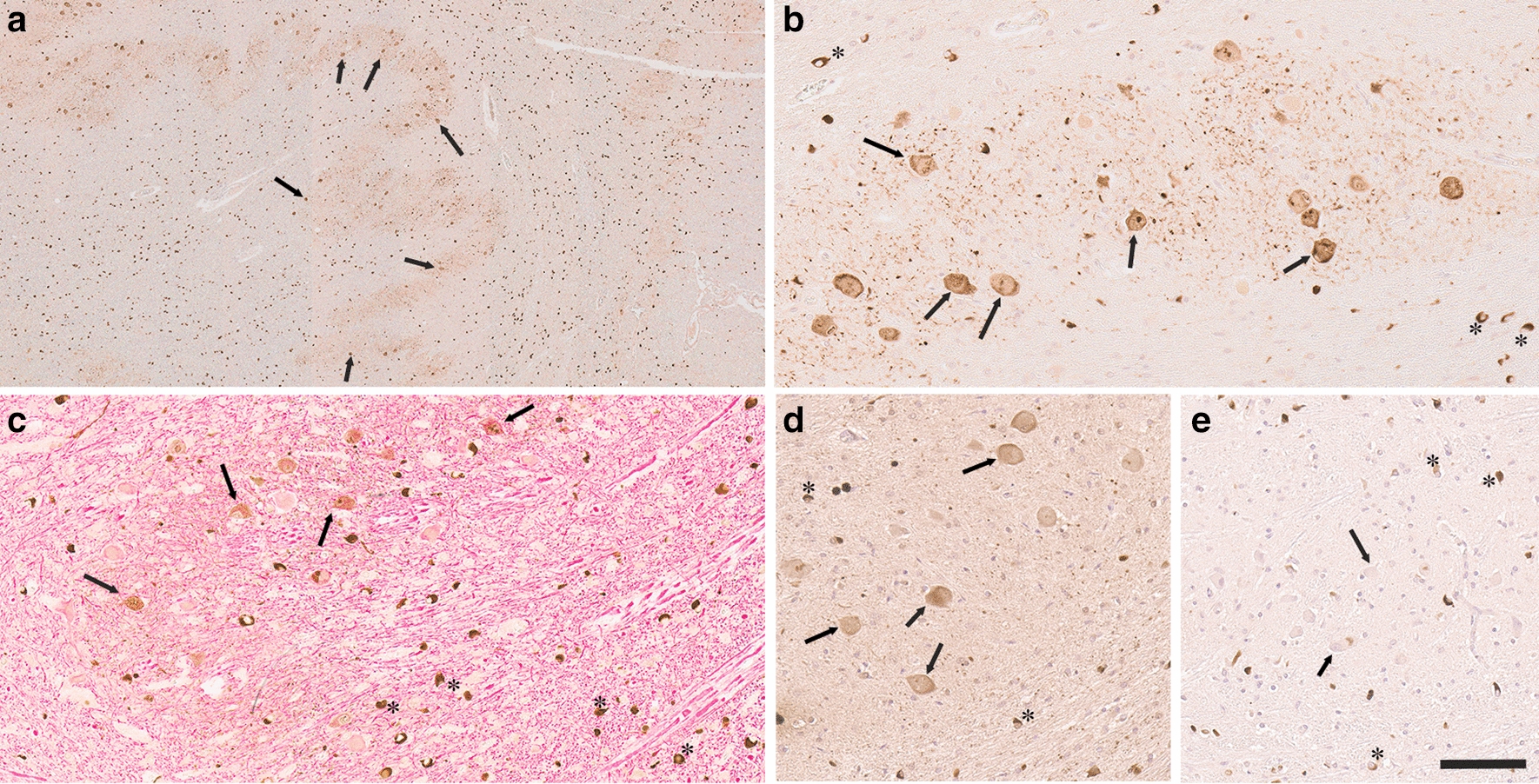
Abundant neuronal inclusions in the inferior olivary nucleus in MSA patients. **a** Low and **b** higher magnification images of inferior olivary nucleus region stained with 5H12 antibody showing abundant αSyn inclusion pathology in the cell bodies and nucleus surrounded by extensive neuritic pathology. **c** Double labeling with anti-NFL (red) and anti-αSyn 5H12 (brown) antibodies in the inferior olivary nucleus region. **d** Perikaryal inclusions within the inferior olivary nucleus are also labeled by anti-ubiquitin staining. **e** IHC with anti-p62 antibody 5G3 in the inferior olivary nucleus. In **a**, **b**, **d** and **e**, the tissue sections were also counterstained with hematoxylin. Scale bar = 500 μm in A; 100 μm in **b**–**e**. Arrows depict neuronal inclusions within the inferior olivary nucleus and asterisks highlight GCIs

**Fig. 5:**
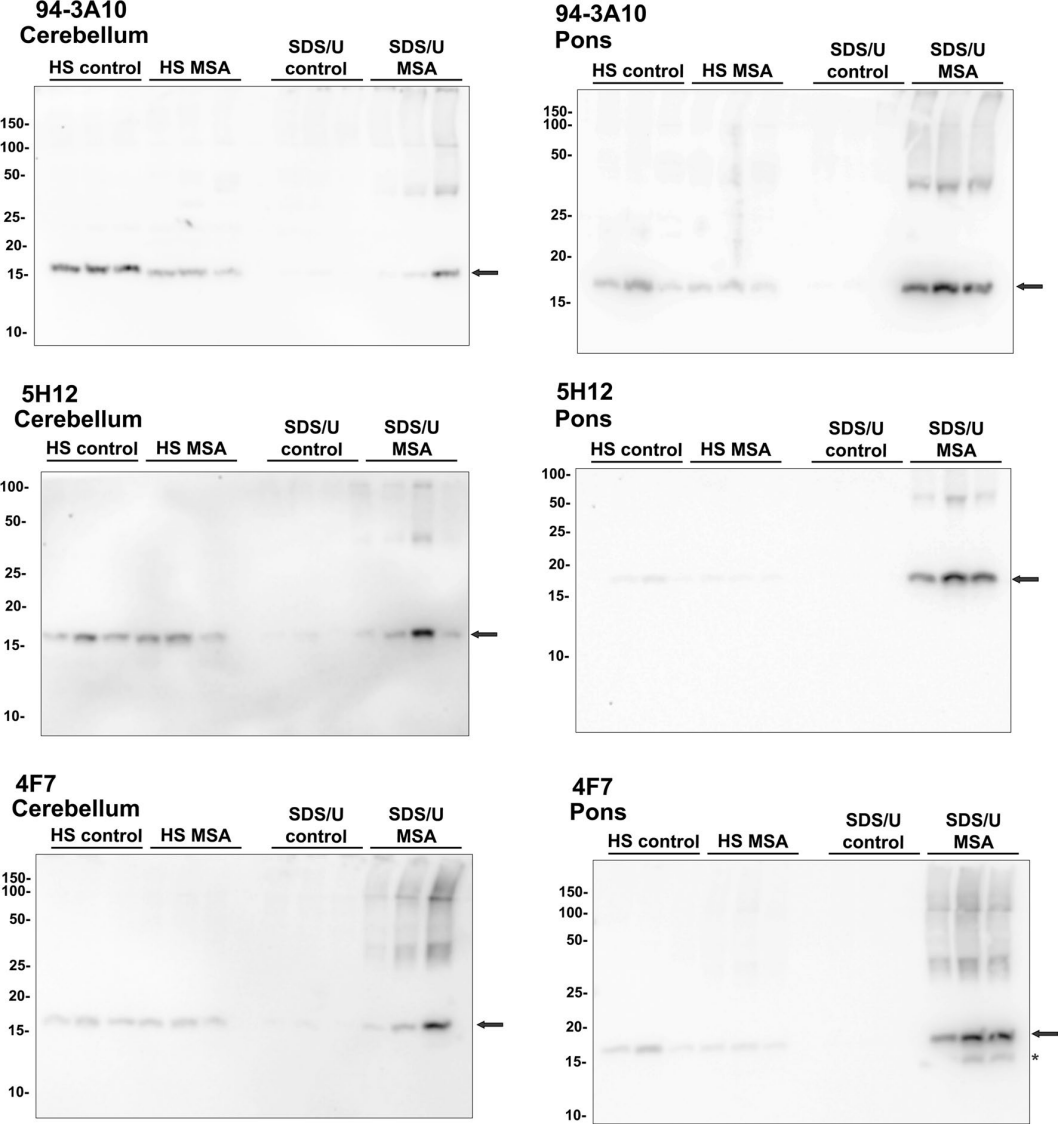
Biochemical fractionation followed by Western blot analysis of cerebellum and pons white matter from control and MSA patients. Equal amounts of protein (10 μg) were loaded in each lane of 15% polyacrylamide gels and proteins were resolved by SDS-PAGE and analyzed by immunoblotting. The HS and SDS/U fractions from the pons and cerebellum of 3 control or MSA patients were analyzed with 94-3A10 (top panels), 5H12 (middle panels) and 4F7 (bottom panels) antibodies, and distinct bands can be seen near ~ 17 kDa (full-length αSyn) in every HS fraction, while only in confirmed cases of MSA in the SDS/U fractions. Additional bands of sizes varying between ~ 30 and ~ 200 kDa can also be seen prominently in the SDS/U fractions of confirmed MSA and not in the controls. Antibody 4F7 detected bands for these same fractions in the pons at a molecular weight lower than 17 kDa. The relative mobility of molecular mass markers is indicated on the left of each blot. Arrows point to full-length αSyn and asterisk indicates carboxy-truncated αSyn

The neuronal cytoplasmic inclusions observed in MSA pontine nuclei were varied in morphology, ranging from aggregates that filled most of the cytoplasm to more defined skein-like inclusions (Fig. 2a–c). The abundant αSyn accumulations stained within processes in the pontine nuclei were not observed in pontine transverse fibers that were laden with GCIs (Fig. 2d), indicating that immunoreactive processes in pontine nuclei are likely dendrites from the pontine nuclei neurons. Further neuroanatomical investigation for other pathologies with the new αSyn antibodies also uniquely revealed abundant neuroaxonal spheroids within the middle cerebral peduncle (Fig. 2e) that are likely the axonal projections of neurons within the pontine nuclei. In the cerebellar hemispheres only GCIs were labelled within the white matter with some additional, less abundant GCIs interspersed within the granular layer (Fig. 2f).

Double staining of the pons with anti-MAP2 or anti-NFL antibodies as neuronal markers and anti-αSyn 5H12 antibody, demonstrated that the αSyn inclusions in pontine nuclei are in neurons with GCIs in close proximity, but not labelled with, the neuronal markers (Fig. 3a, b). IHC with anti-ubiquitin and anti-p62 antibodies revealed major differences between the neuronal inclusions and GCIs (Fig. 3c, d). While GCIs are strongly labeled for ubiquitin and p62, the majority of the neuronal inclusions in the pontine nuclei were not reactive or weakly labeled.

In addition to the pontine nuclei, abundant neuronal αSyn inclusion pathology was also observed in the inferior olivary nucleus of the medulla oblongata, and can be readily identified, even at low magnifications (Fig. 4a). These inclusions were not observed in control cases (Additional file 1: Fig. S4) and filled most of the neuronal cell bodies, with aggregates often in the nucleus (Fig. 4b). The neurites surrounding these neurons also contained extensive αSyn accumulation that can be discerned, even at low magnification (Fig. 4a, b). The neuronal inclusions in the inferior olivary nucleus were double labeled for anti-NFL (Fig. 4c). These neuronal inclusions were modestly labeled for ubiquitin while mostly not reactive for p62 compared to GCIs that were strongly labeled for both ubiquitin and p62 (Fig. 4d, e).

To further characterize the αSyn inclusions present in MSA patients, white matter tissue from the cerebellar hemispheres that predominantly contained GCIs and the pons containing the pontine nuclei with both neuronal inclusions and GCIs were sequentially biochemically fractionated with solution of increased protein solubility (Additional file 1: Fig. S6) and analyzed by immunoblotting (Fig. 5). The MSA fractions were compared to extracts from control patients with 3 different antibodies: 4F7 (epitope residues 106–115), 5H12 (epitope residues 113–122) and 94-3A10 (epitope residues 130–140). In control patients, αSyn was detected in the HS soluble fractions which was also detected in MSA patients, but with a redistribution into the SDS/U fractions that reflects protein aggregation in MSA patients (Fig. 5). αSyn was mostly present at the molecular mass of full-length protein in the HS soluble fractions in control and MSA fractions for both brain regions. In the SDS/U fractions from the cerebellum of MSA patients, full-length αSyn was detected, as well as higher molecular mass smear that represent modified forms of αSyn, especially with antibody 4F7. In the pons of MSA patients, similar findings were observed, but with additional carboxy-truncated αSyn that was detected with antibody 4F7 (Fig. 5).

**Fig. 6:**
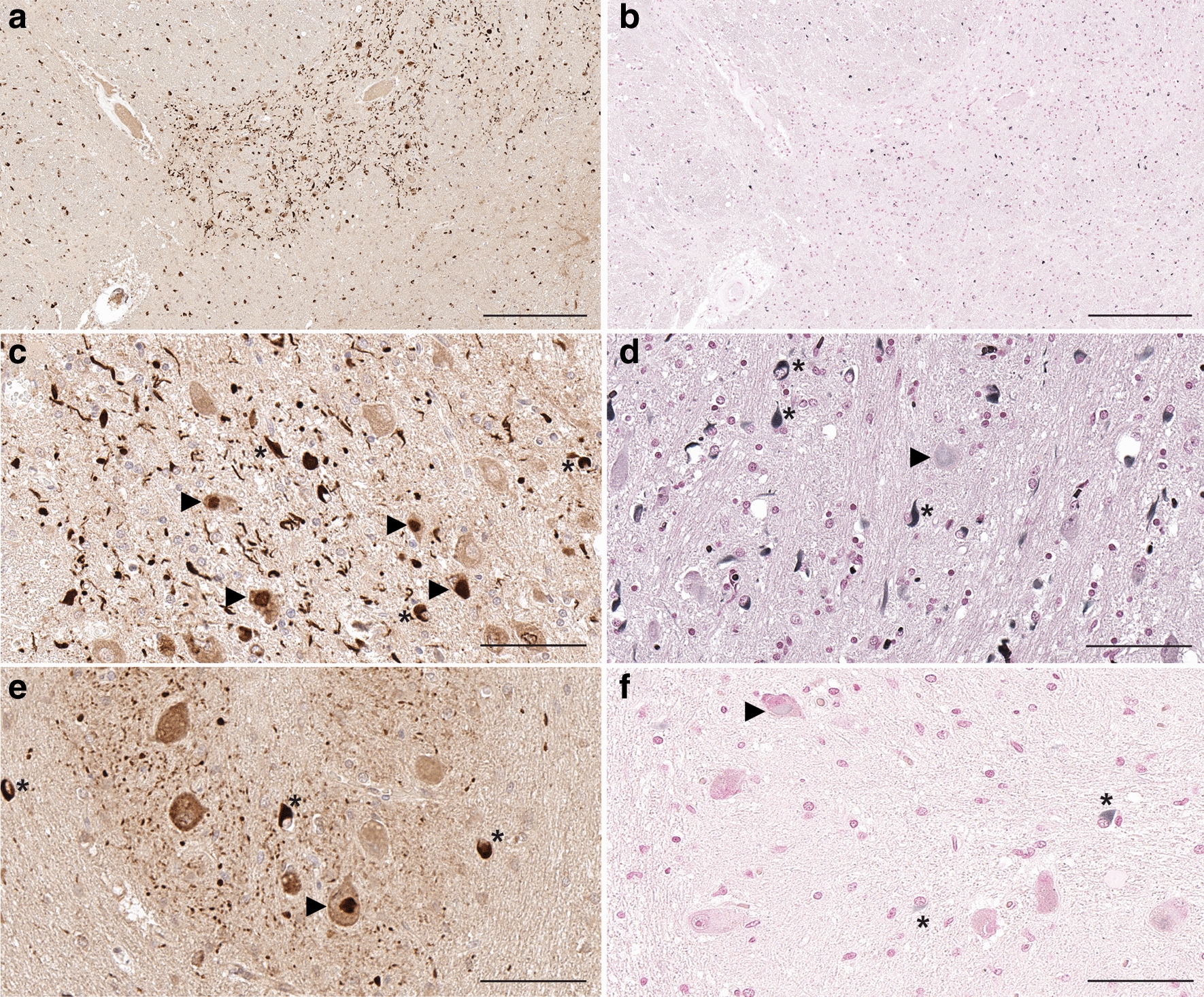
GCIs and brainstem neuronal inclusions are differentially reactive to Gallyas silver staining. **a** Low magnification images of pontine nuclei demonstrate abundant labeling of GCIs, neuritic pathology and neuronal inclusions with 5H12 antibody, **b** while only a subset of GCIs and rare neuritic pathology are highlighted by Gallyas silver stain. **c**, **d** High magnification images of pontine nuclei and **e**, **f** inferior olivary nucleus underscore labeling of GCIs, neuritic pathology and neuronal inclusions with 5H12 antibody **c**, **e**. Gallyas silver staining only reveals a subset of GCIs and rare neuritic pathology in pontine nuclei and neurons of the inferior olive **d**, **f**. Rare neuronal inclusions show faint labeling with Gallyas silver staining (arrowheads, **d**, **f**). Asterisks highlight GCIs. Scale bar: 300 μm in **a** and **b**, 60 μm in **c**–**f**

As GCIs were initially discovered using Gallyas silver staining that differentiates them from Lewy pathology [3, 38], we compared anti-αSyn 5H12 staining with Gallyas silver staining. While 5H12 antibody readily highlighted abundant GCIs, neurites, and neuronal inclusions in the  pontine nuclei (Fig. 6a, c) and inferior olivary nucleus (Fig. 6e), Gallyas silver staining predominantly labeled GCIs (Fig. 6b, d, f), while neuronal inclusions only showed occasional faint labeling (Fig. 6d, f, arrowheads) and neurites were largely negative. We also noted that only a subset of GCIs were silver positive, while 5H12 antibody staining highlighted these inclusions in much higher abundancy (Fig. 6c-f).

## Discussion

Graham and Oppenheimer [39] originally proposed that adult patients with MSA clinical symptoms should be classified as one disorder, but this nosology was not unequivocally established until GCIs were discovered as the hallmark defining inclusion pathology of MSA [3]. These inclusions are predominantly comprised of αSyn abnormally polymerized into fibrils [4, 5] and a definite diagnosis of MSA still requires a post-mortem assessment for the presence of GCIs [1, 2].

MSA is one of many α-synucleinopathies where the progressive accumulation of αSyn pathological inclusions in the brain is associated with the insidious nature of these diseases [18, 19]. A major pathological difference between MSA and other α-synucleinopathies is the extensive accumulation of αSyn in oligodendrocytes [6, 7, 9–11, 14], while in other neurodegenerative diseases αSyn inclusions are predominantly neuronal [18, 19]. Since αSyn is an abundant and widely expressed neuronal brain protein [20–23], the presence of neuronal αSyn inclusions is not unexpected, although the specific vulnerability of affected neuronal populations is still not completely explained [18, 19, 40]. Even more enigmatic has been the source of αSyn in MSA GCIs, and the initial driver of αSyn pathology, as oligodendrocytes express marginal levels of αSyn [26].

Many experimental findings have supported the notion that αSyn pathology can spread throughout the CNS by a prion-like conformational templating mechanism [19, 24, 25]. MSA typically has a more rapid clinical progression than other Lewy body diseases [1, 19, 41] suggesting a  more aggressive spread of pathology by MSA-associated αSyn prions. Congruent with this notion, many experimental findings indicate that GCIs are comprised of polymerized αSyn into distinct prion strains with greater seeding activity compared to preparations derived from neuronal Lewy inclusions. Initial seeding studies in animals and many subsequent investigations using MSA extracts for transmission in mice were performed using M83 A53T αSyn transgenic mice, which are quite permissive to the extensive induction of CNS αSyn pathology, resulting in a lethal motor phenotype following intracellular inoculation compared to other αSyn transgenic models [42–46]. Using the M83 αSyn transgenic mice, CNS αSyn pathology can also be induced by peripheral inoculation with MSA extracts [47]. Thereafter, it was also determined that detergent insoluble MSA extracts, enriched in aggregated αSyn, can also induce αSyn pathology when injected in the brain of wild non-transgenic mice [48, 49]. Using either αSyn transgenic mice or wild type mice, it was shown that aggregated αSyn within lysates from MSA patient brains are significantly more potent at inducing inclusion pathology than similar lysates from Parkinson’s disease or LBD patients [43, 48–50]; however, the prominent induced form of pathology, when inoculating mice with MSA extracts, is consistently neuronal and not in oligodendrocytes [42–44, 48–50]. The specific induction of αSyn pathology in oligodendrocytes was only observed in mice that transgenically overexpress human αSyn in oligodendrocytes in a mouse αSyn null background [48]. Using αSyn seeds comprised of recombinant αSyn proteins to induce brain pathology in wild type mice can result in some rare inclusions in oligodendrocytes but these are much less abundant than the induced neuronal pathology and they take much longer time to accumulate [51]. The greater potency of the MSA αSyn prion strain compared to Lewy type in Parkinson’s disease or LBD also has been observed in cultured cells seeding studies [43, 48, 52, 53].

We report here at least 2 major neuronal populations affected in MSA—the pontine nuclei and inferior olivary nucleus, which contain much more neuronal αSyn pathology than previously appreciated—although neuronal αSyn inclusions have been observed to varying degrees within various brain regions in previous studies [6–16, 54]. This abundant neuronal αSyn inclusion pathology in MSA  was revealed using a new series of αSyn antibodies targeting the carboxy-terminus of αSyn. It was previously demonstrated that staining of GCIs versus Lewy bodies, and even labeling within a given population of GCIs with different αSyn antibodies, can be variable, consistent with the presence of different conformers of polymerized αSyn, akin to different prion-like strains with different degrees of epitope presentation [9, 11, 48, 54]. These previous studies align with the notion that the new αSyn antibodies described are more effective at detecting αSyn strains that were concealed in these neuronal populations affected by MSA. Recent detailed cyro-EM molecular analysis of MSA αSyn fibrils clearly demonstrated that these have different structures than those from LBD or those assembled from recombinant proteins [55]. Even within MSA αSyn fibrils, many different conformers exist [55] suggesting the likelihood of completely independent and diverse αSyn prion strains or related sub-strains within a given brain. The new antibodies described here are likely more sensitive in revealing some of these subpopulations of aggregated αSyn with conformations that might mask some epitopes.

The extensive neuronal αSyn inclusions in MSA pontine nuclei and inferior olivary nucleus have distinct properties compared to GCIs. They are only weakly reactive to ubiquitin and p62 IHC, as well as Gallyas silver staining compared to GCIs. Our biochemical analyses of MSA brain tissue followed by immunoblotting revealed the presence of aggregated αSyn in both the cerebellar white matter, which contains predominantly GCIs, and the pons, but these presented region differentiating signatures. In the pons, there is a clear accumulation of αSyn carboxy-truncated between residues 115 and 122. Although at this time we cannot determine if these are specifically associated with the neuronal inclusions in the pons, we are currently characterizing novel antibodies specific for carboxy-truncated forms of αSyn to address this issue in future studies.

Given that the pathophysiology underlying the formation of GCIs in MSA is still enigmatic, as oligodendrocytes express marginal levels of αSyn, we propose that the neuronal αSyn inclusion pathology revealed here could be a major contributor to GCI formation. Oligodendrocytes clearly have the ability to take up various forms of αSyn [56]. Besides the neuronal populations identified here, additional neuronal populations with copious amount of aggregated αSyn, potentially produce seeds that are preferentially taken up by oligodendrocytes and are altered into strains with high affinity for oligodendrocytes. Some experimental modeling studies in mice and in cultured cells implicate that the intracellular environmental milieu of the oligodendrocytes is a driving factor in producing a GCI-specific αSyn prion strain(s) which has higher infectivity [48]. Nevertheless, in these studies αSyn strains generated in oligodendrocytes did not present cell type-specific infectivity, although the intrinsic greater infectivity generated the oligodendrocyte environment could be passaged [48]. However, these studies were performed using murine cells and it is possible that oligodendrocyte-specific αSyn prion strains are generated in a human cellular environment. This type of oligodendrocyte-mediated strain would acquire the properties of GCIs in terms of ubiquitin, p62 and Gallyas silver reactivity. The notion that human MSA GCI-type αSyn strains are selectively generated in a human cellular environment would be consistent with the findings that MSA transmission seeding studies in mice did not induce pathology that was Gallyas silver reactive [42, 50], although the induced pathology was also predominantly neuronal.

Furthermore, as GCI burden correlates with degeneration of some neuronal populations [57, 58], it is possible that αSyn GCI-type prion strains were also taken up by some neuronal populations, but that these are particularly toxic to some neurons, such that αSyn neuronal inclusions that might have existed during the course of the disease are not observed at end stage. This hypothesis would imply that the neurons in the pontine nuclei and inferior olivary nucleus might be more resistant to such strains of misfolded αSyn and that these neurons therefore may also act as a more permanent reservoir of seeds.

Most of the MSA tissues used for the reported seeding studies were from basal ganglia, midbrain, and cerebellum regions that contained predominantly GCIs and only sparse neuronal αSyn inclusions [42–47, 49, 50]. We are not aware of any seeding studies that used medulla that would comprise the abundant neuronal inclusions described here, but a limited number of experiments have used pontine tissue [43] that might have contained the neuronal inclusions underscored here. Future studies should take advantage of the new antibodies described here to re-examine the abundance of neuronal αSyn inclusions in human tissue used for transmission studies and to design seeding studies from MSA brain regions that contain abundant neuronal inclusions compared to almost exclusively GCIs; acknowledging that comparative studies of tissues with only one type of inclusion will be very difficult, as GCIs are interspersed in close proximity of the neuronal inclusions in the pontine nuclei and inferior olivary nucleus.

## Conclusions

Taking advantage of a novel series of αSyn antibodies targeting the carboxy-terminal region, our studies underscore the abundance of αSyn inclusion pathology in the pontine nuclei and inferior olivary nucleus of MSA patients. These neuronal αSyn inclusions are characterized by distinct histological properties relative to GCIs. It is possible that this abundant reservoir of aberrant αSyn could be a primary source of αSyn prion seeds that drives MSA pathobiology, but this notion will have to be further experimentally investigated in future studies.

## Supplementary Information

**Additional file 1**: **Supplementary Fig. 1.** Location of the epitopes for the new αSyn antibodies. The amino acid sequences of human and mouse αSyn, as well as human γSyn and human βsyn, are shown with points of non-homology to human αSyn highlighted. The three regions of αSyn (N-terminus, NAC region and C-terminus) are labeled and displayed above the sequences. The epitopes of the five novel C-terminal αSyn antibodies were determined based on the peptide used for immunization and immunoblot analysis using carboxy--truncated recombinant αSyn protein (see Additional file 1: Supplemental Fig. 5). **Supplementary Fig. 2.** IHC staining of tissue sections from LBD patients in the substantia nigra, cingulate cortex and amygdala with the various αSyn antibodies labeling Lewy bodies and Lewy neurites. Tissue sections were stained with the αSyn antibodies indicated in the top of each column. All sections were counterstained with hematoxylin. Scale bar = 100 μm. **Supplementary Fig. 3.** IHC staining of cerebellum tissue sections from an MSA patient with the various αSyn antibodies labeling GCIs. Tissue sections were stained with the αSyn antibodies indicated in the top left corner. All sections were counterstained with hematoxylin. Scale bar = 100 μm. **Supplementary Fig. 4.** IHC staining with αSyn antibody 5H12 in a control patient. Tissue sections from **a** the cerebellum, **b** pons and **c** medulla oblongata showing the paucity of pathological inclusions. Sections were counterstained with hematoxylin. Scale bar = 300 μm. **Supplementary Fig. 5.** Western blot analysis to refine the epitope map and characterize the specificity of the new αSyn antibodies. Immunoblot analysis using recombinant human γSyn, human βSyn, mouse (m) αSyn, human (h) αSyn and a series of carboxy-truncated human αSyn (1–129, 1–125, 1–122, 1–119 and 1–115) proteins. 210 ng of each protein was loaded per lane and membranes were probed with the antibodies labeled above. The relative mobility of molecular mass markers is indicated on the left of each blot. **Supplementary Fig. 6.** Schematic for biochemical fractionation of human brain tissue. Horizontal arrows indicate the solutions added to either the homogenized tissue or pellet resulting from the previous step. From top to bottom, the collected fractions were high salt, HS/Triton X-100 fraction radioimmuno-precipitation assay (RIPA) and SDS/urea fractions. (PDF 11290 kb)

## Data Availability

All data generated or analyzed during this study are included in this published article and its supplementary information files.

## Abbreviations

αSyn: α-Synuclein
βSyn: β-Synuclein
BSA: Bovine serum albumin
DAB: 3,3′-Diaminobenzidine
ELISA: Enzyme-linked immunosorbent assay
FBS: Fetal bovine serum
γSyn: γ-Synuclein
GCI: Glial cytoplasmic inclusion
HS: High salt
HS/T: High salt/Triton X-100
IHC: Immunohistochemistry
LBD: Lewy body dementia
MAP2: Microtubule-associated protein 2
mcKLH: Mariculture keyhole limpet hemocyanin
MSA: Multiple system atrophy
MSA-C: Multiple system atrophy predominant cerebellar-type
MSA-P: Multiple system atrophy predominant parkinsonian-type
NFL: Neurofilament light chain
p62: P62/sequestrosome-1
RIPA: Radioimmunoprecipitation assay
SDS/U: SDS urea
TBS: Tris-buffered saline
