# Supplementary material for: Robust α-synuclein pathology in select brainstem neuronal populations is a potential instigator of multiple system atrophy
Source: Acta Neuropathol Commun. 2021 May 3;9:80. doi: 10.1186/s40478-021-01173-y (PMC8091528; doi:10.1186/s40478-021-01173-y)
Supplement: Supplementary file 1 — Additional file 1: Supplementary Fig. 1. Location of the epitopes for the new αSyn antibodies. The amino acid sequences of human and mouse αSyn, as well as human γSyn and human βsyn, are shown with points of non-homology to human αSyn highlighted. The three regions of αSyn (N-terminus, NAC region and C-terminus) are labeled and displayed above the sequences. The epitopes of the five novel C-terminal αSyn antibodies were determined based on the peptide used for immunization and immunoblot analysis using carboxy--truncated recombinant αSyn protein (see Additional file 1: Supplemental Fig. 5). Supplementary Fig. 2. IHC staining of tissue sections from LBD patients in the substantia nigra, cingulate cortex and amygdala with the various αSyn antibodies labeling Lewy bodies and Lewy neurites. Tissue sections were stained with the αSyn antibodies indicated in the top of each column. All sections were counterstained with hematoxylin. Scale bar = 100 μm. Supplementary Fig. 3. IHC staining of cerebellum tissue sections from an MSA patient with the various αSyn antibodies labeling GCIs. Tissue sections were stained with the αSyn antibodies indicated in the top left corner. All sections were counterstained with hematoxylin. Scale bar = 100 μm. Supplementary Fig. 4. IHC staining with αSyn antibody 5H12 in a control patient. Tissue sections from a the cerebellum, b pons and c medulla oblongata showing the paucity of pathological inclusions. Sections were counterstained with hematoxylin. Scale bar = 300 μm. Supplementary Fig. 5. Western blot analysis to refine the epitope map and characterize the specificity of the new αSyn antibodies. Immunoblot analysis using recombinant human γSyn, human βSyn, mouse (m) αSyn, human (h) αSyn and a series of carboxy-truncated human αSyn (1–129, 1–125, 1–122, 1–119 and 1–115) proteins. 210 ng of each protein was loaded per lane and membranes were probed with the antibodies labeled above. The relative mobility of molecular mass markers is indicated [file 40478_2021_1173_MOESM1_ESM.pdf]

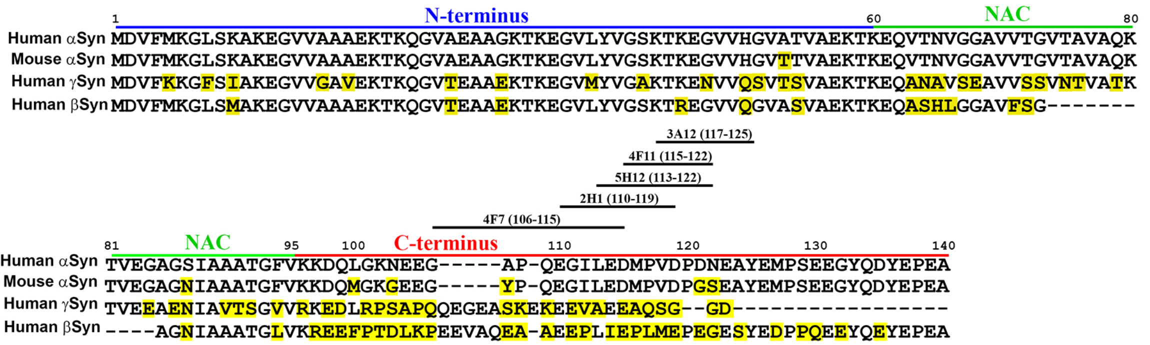

Supplemental Figure 1

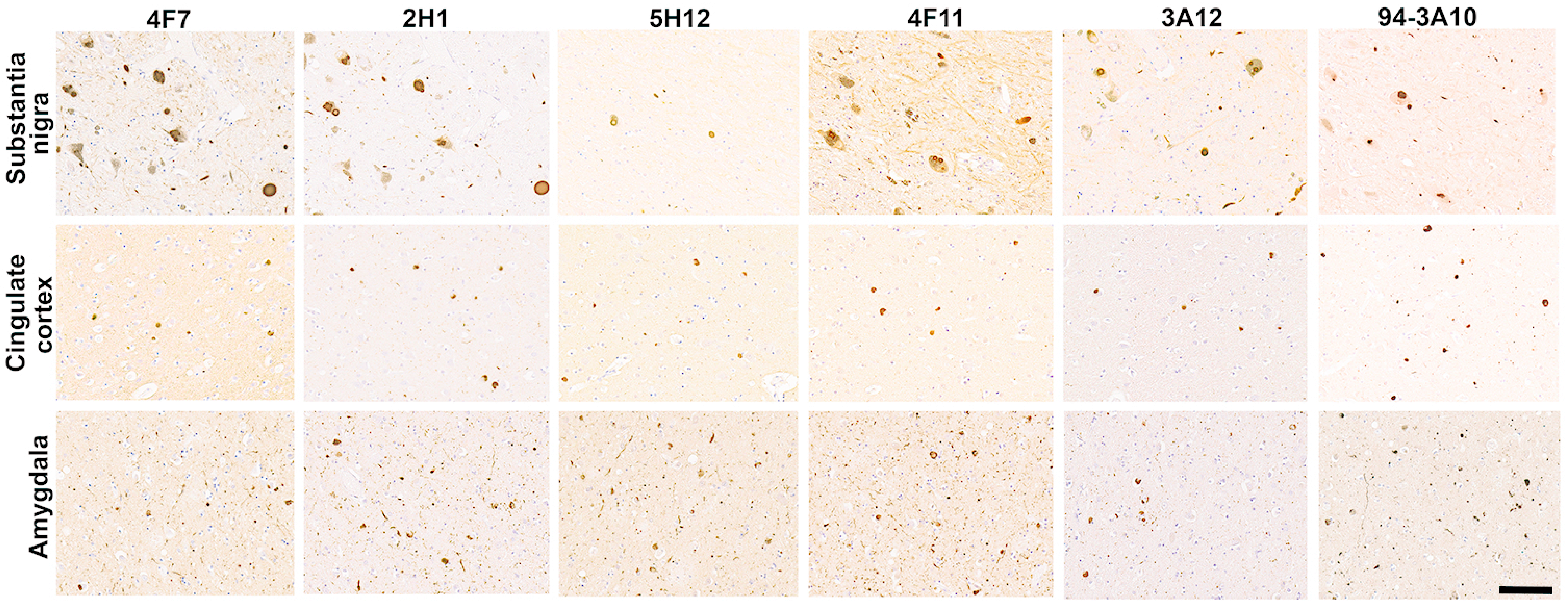

Supplemental Figure 2

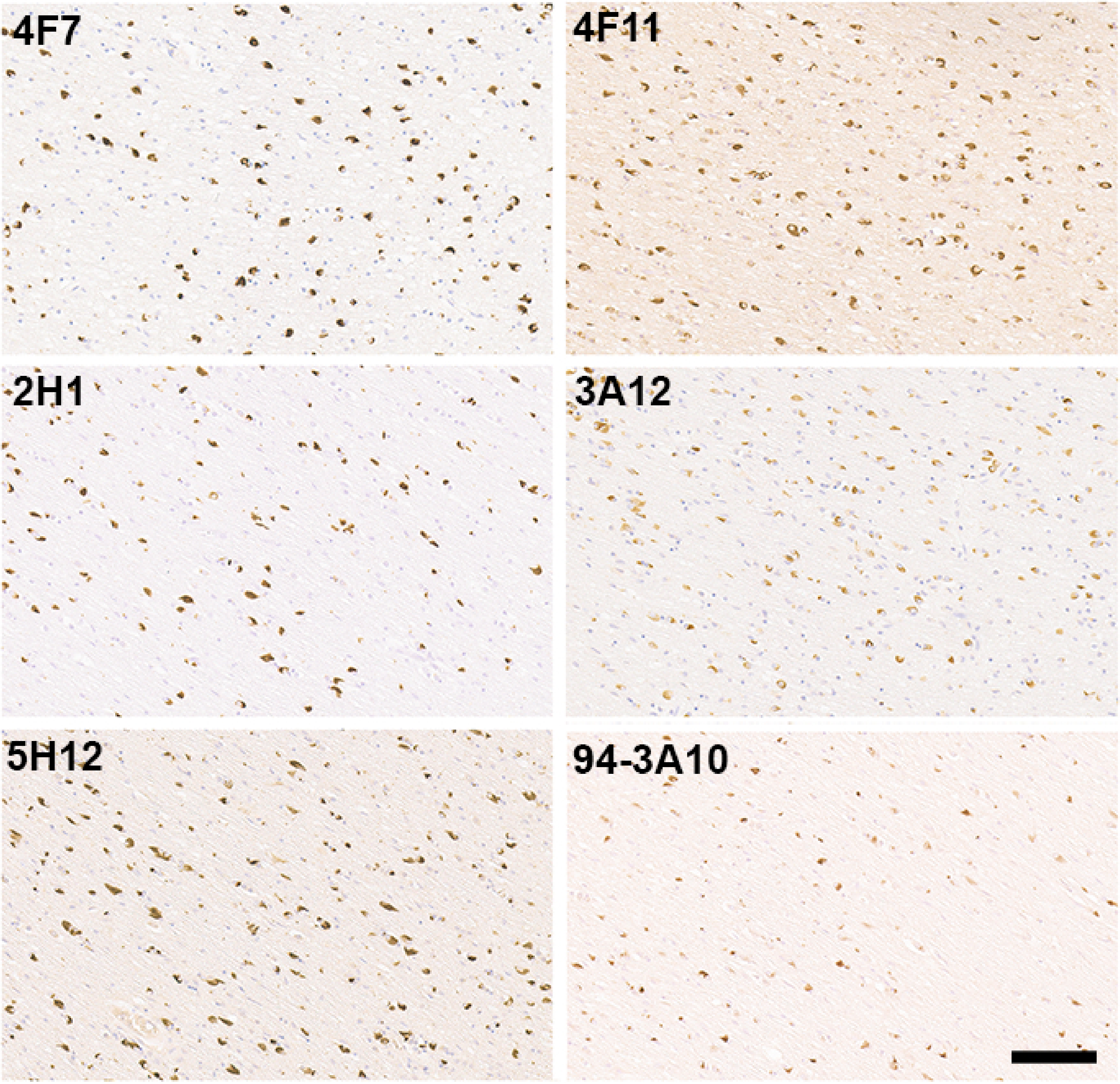

Supplemental Figure 3

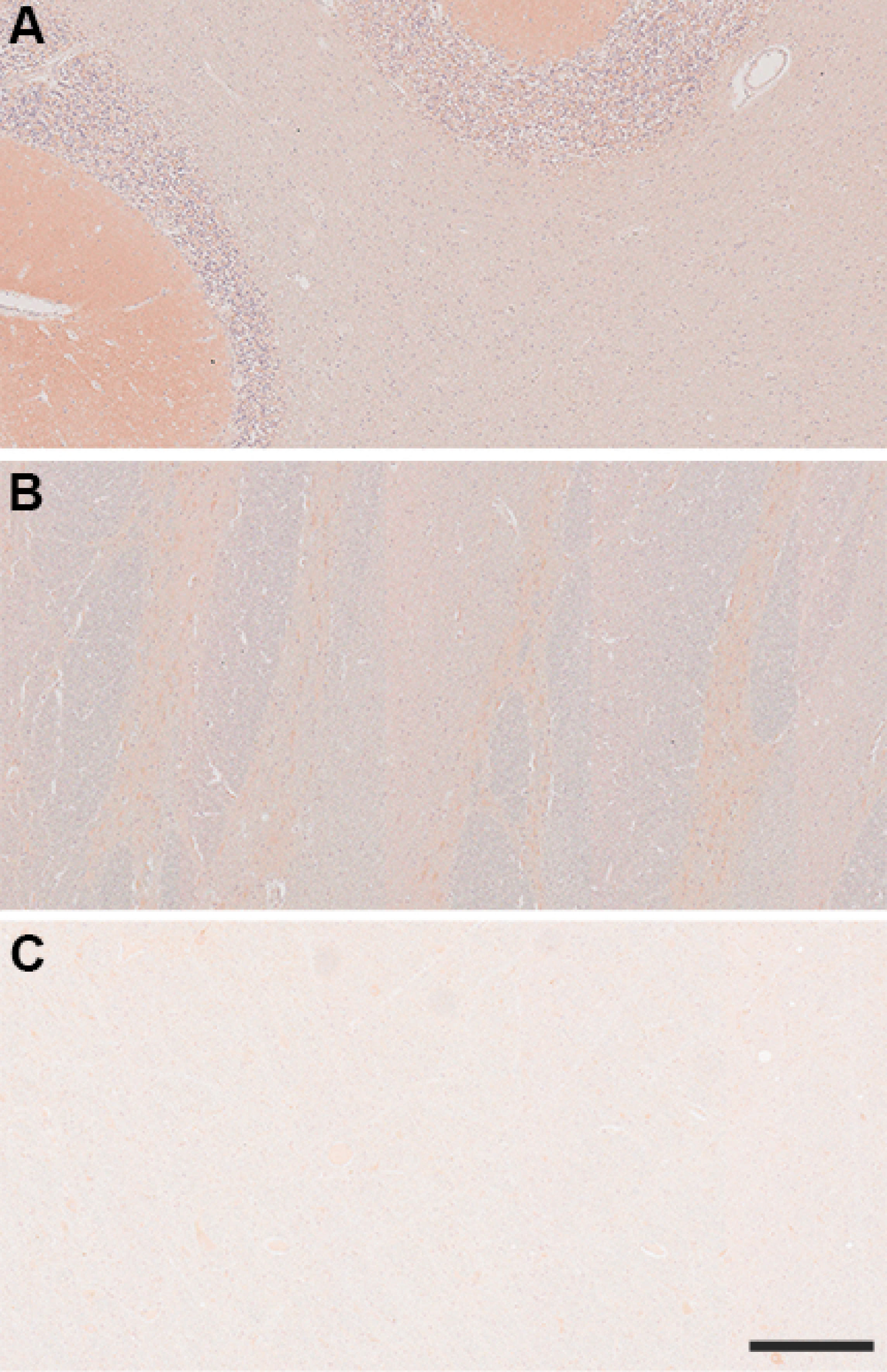

Supplemental Figure 4

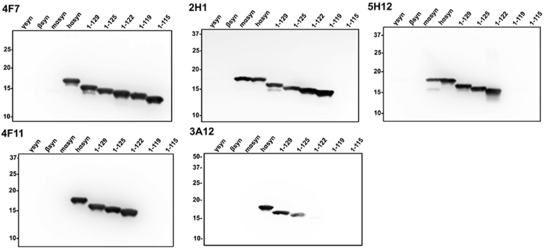

Supplemental Figure 5

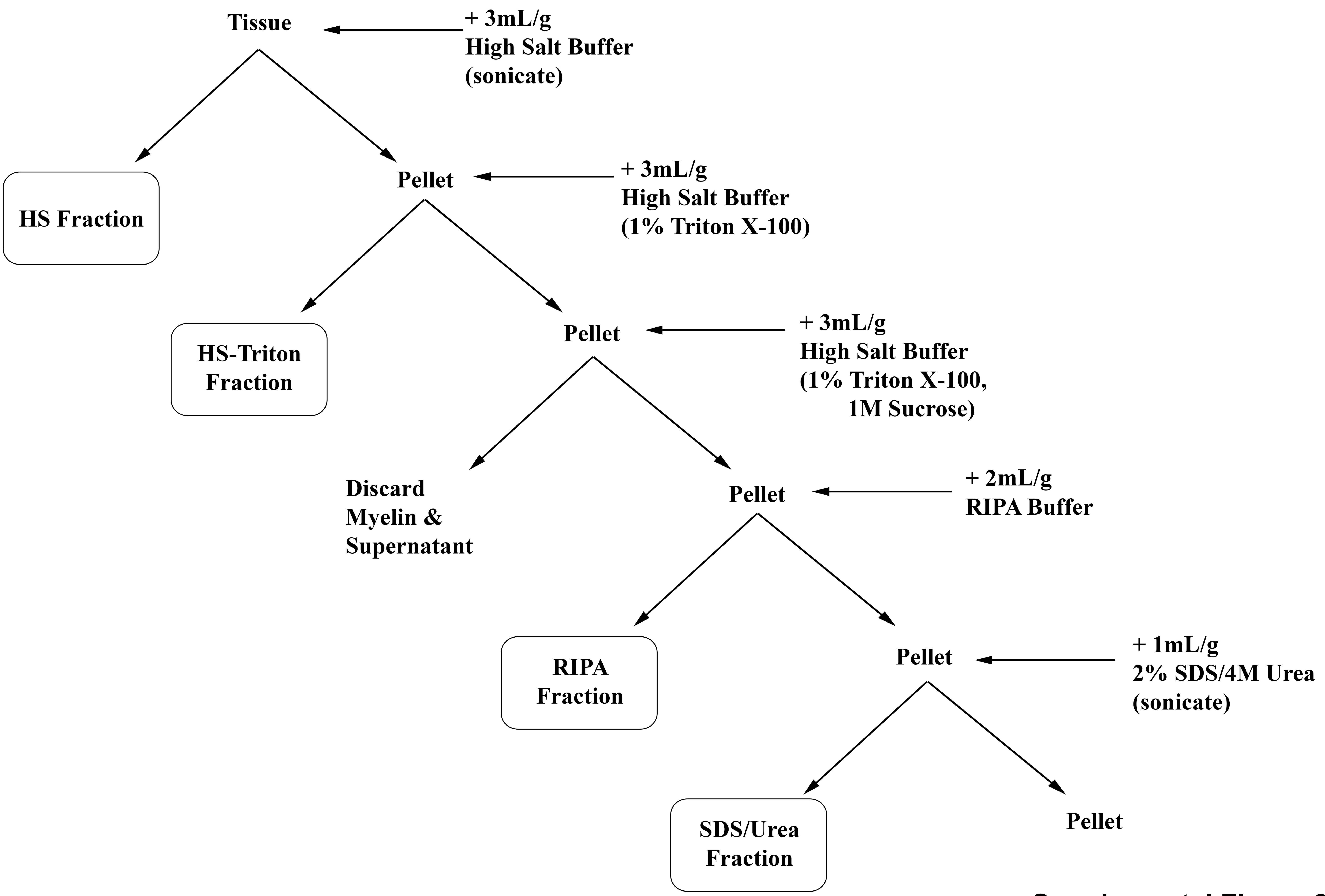

Supplemental Figure 6
